# EphA2 promotes the transcription of KLF4 to facilitate stemness in oral squamous cell carcinoma

**DOI:** 10.1007/s00018-024-05325-w

**Published:** 2024-06-25

**Authors:** Junqiang Bai, Yang Chen, Yunqing Sun, Xinmiao Wang, Yifan Wang, Shutian Guo, Zhengjun Shang, Zhe Shao

**Affiliations:** 1 State Key Laboratory of Oral & Maxillofacial Reconstruction and Regeneration, Key Laboratory of Oral Biomedicine Ministry of Education, Hubei Key Laboratory of Stomatology, School & Hospital of Stomatology, Wuhan University, Wuhan, China; 2 Department of Oral and Maxillofacial Surgery, School & Hospital of Stomatology, Wuhan University, Wuhan, China; 3 Department of Oral and Maxillofacial-Head and Neck Oncology, School & Hospital of Stomatology, Wuhan University, Wuhan, China; 4 Day Surgery Center, School and Hospital of Stomatology, Wuhan University, Wuhan, China

**Keywords:** OSCC, Cancer stem cells, EphA2, KLF4, YAP

## Abstract

Ephrin receptor A2 (EphA2), a member of the Ephrin receptor family, is closely related to the progression of oral squamous cell carcinoma (OSCC). Cancer stem cells (CSCs) play essential roles in OSCC development and occurrence. The underlying mechanisms between EphA2 and CSCs, however, are not yet fully understood. Here, we found that EphA2 was overexpressed in OSCC tissues and was associated with poor prognosis. Knockdown of EphA2 dampened the CSC phenotype and the tumour-initiating frequency of OSCC cells. Crucially, the effects of EphA2 on the CSC phenotype relied on KLF4, a key transcription factor for CSCs. Mechanistically, EphA2 activated the ERK signalling pathway, promoting the nuclear translocation of YAP. Subsequently, YAP was bound to TEAD3, leading to the transcription of KLF4. Overall, our findings revealed that EphA2 can enhance the stemness of OSCC cells, and this study identified the EphA2/KLF4 axis as a potential target for treating OSCC.

**Supplementary Information:**

The online version contains supplementary material available at 10.1007/s00018-024-05325-w.

## Introduction

Oral squamous cell carcinoma (OSCC) arises from the floor of the mouth, buccal mucosa, tongue, and other areas inside the oral cavity and is the most prevalent subtype of head and neck squamous cell carcinoma [1]. According to the Global Cancer Observatory, there were 377,713 new cases of OSCC and 177,757 related deaths worldwide in 2020, with both deaths and new cases increasing compared to those in 2018 [2, 3]. The classic treatments for OSCC include surgery, chemotherapy, radiotherapy, or a combination of these treatments, depending on the clinical condition of the patients [4]. Although other therapies are being explored and some have even been applied, the 5-year survival rate of patients with OSCC remains unsatisfactory [5, 6], which is largely attributable to cancer recurrence, metastasis, and treatment resistance [7, 8].

Cancer stem cells (CSCs), which are defined as cells capable of asymmetric division and tumour-initiating abilities, play a fundamental role in therapeutic resistance, recurrence, and metastasis [9–12]. Therefore, targeting CSCs is a promising therapy for various tumours, including OSCC [13–15]. However, the clinical therapeutic efficacy for eliminating CSCs is unsatisfactory at present. One of the reasons is that the mechanisms regulating CSCs have not been fully revealed. Several studies have identified transcription factors (TFs), such as KLF4, SOX2, c-MYC, and OCT4, as key determinants of CSCs [11, 16]. For example, KLF4 overexpression reversed the MED27 silencing-mediated arrest of stemness maintenance capacity in breast cancer in vitro and in vivo [17], and upregulation of SOX2 promoted CSC properties in prostate cancer [18]. These studies suggest that regulating these key TFs may be effective in eliminating CSCs and increasing the effectiveness of cancer treatment.

Krüppel-like Factor 4 (KLF4) is a prominent member of the evolutionarily conserved Krüppel-like factor family of zinc finger transcription factors [19]. KLF4 is well known for its role in sustaining embryonic stem cell stemness and inducing pluripotent stem cell formation [20, 21]. Interestingly, KLF4 has also been shown to play a role in maintaining the stemness of CSCs; for instance, KLF4-overexpressing osteosarcoma cells displayed characteristics of osteosarcoma CSCs: increased tumour sphere-forming potential, enhanced levels of stemness-associated genes, and great chemoresistance to adriamycin and CDDP [22]. However, its role in OSCC CSCs remains unclear.

Ephrin receptor A2 (EphA2), a 130 kDa transmembrane glycoprotein, is a member of the Ephrin receptor family, which is the most famous class of receptor tyrosine kinases (RTKs) [23]. Although the role of other members of the Eph family has also been explored in tumours, EphA2 is extremely arresting [24]. Many studies have demonstrated that it can promote proliferation, survival, angiogenesis, invasion, migration, metastasis, and stemness in cancer [24–26]. Our previous study also showed that EphA2 overexpression is associated with poor prognosis in OSCC patients [27]. However, the specific mechanism of EphA2 in OSCC and whether EphA2 is associated with CSCs are unknown.

In this work, we found that EphA2 promoted the stemness of OSCC cells and served as a promising therapeutic target in OSCC. We found that EphA2 was highly expressed in OSCC tissues and contributed to poor survival. Furthermore, after EphA2 knockdown, the proliferation, self-renewal, and sphere-forming capacity of OSCC cells were significantly impaired. Mechanistically, EphA2, by evoking the ERK pathway, promoted the nuclear translocation of Yes-associated protein (YAP). Subsequently, YAP bound to TEA domain family protein 3 (TEAD3) and enhanced the transcription of the critical CSC-related TF KLF4, resulting in increased expression of the CSC-related markers CD44 and CD133. In addition, EphA2 knockdown inhibited the growth and stemness of OSCC cells in vivo, while overexpression of YAP or KLF4 partly reversed these effects.

## Materials and methods

### Reagents

TBHQ and SCH772984 were purchased from MedChem Express (MCE, NJ, USA) and were dissolved in dimethyl sulfoxide as stock solutions and stored at -80 °C. The solution was diluted in medium to reach the appropriate concentrations.

### Cell lines and culture

The OSCC cell lines SCC4, SCC9, SCC25, and CAL27 were purchased from the China Center for Type Culture Collection (Shanghai, China). CAL27 cells were cultivated in DMEM. SCC4, SCC9 and SCC25 cells were cultivated in DMEM/F-12. Then, 10% FBS was added to the medium.

### Human tissue samples

The human specimens involved in this experiment consisted of two parts: a tissue microarray (58 human OSCC tissues and 12 normal oral mucosa) and sixteen pairs of tissues from the OSCC patient (OSCC tissue and adjacent noncancer tissues). Written informed consent was obtained from all participants. All procedures were in accordance with the Declaration of Helsinki, and the research protocols were approved by the Ethics Committee of the School and Hospital of Stomatology, Wuhan University. For patient information, see Supplementary Tables 1 and 2.

### Cell counting Kit-8 (CCK-8) assay

CCK-8 was purchased from Biosharp (BS350B). In the cell proliferation assay, we inoculated well-grown CAL27 or SCC9 cells into 96-well plates at a dose of 3 × 10^3^ per well, and the cells were cultured in humidified 37 °C, 5% CO2 incubators for 24 h, 48 h, 72 h or 96 h. After the completion of culture at these four time points, cell culture medium (100 µl) containing 10 µl of CCK-8 reagent was added, and the cells were incubated for an additional 2 h. Subsequently, the absorbance at 450 nm was measured. For the cytotoxicity assays, we inoculated well-grown CAL27 or SCC9 cells into 96-well plates at a dose of 5 × 10^3^ cells/well, and the cells were cultured in humidified incubators at 37 °C and 5% CO2 for 24 h. After that, cisplatin at different concentrations (1.25 µM, 2.5 µM, 5 µM, 10 µM, 20 µM, 40 µM and 80 µM) was added. After an additional 24 h of incubation, cell culture medium (100 µl) containing 10 µl of CCK-8 was added, and the cells were incubated for an additional 2 h. Subsequently, the absorbance at 450 nm was measured. The inhibition ratio was calculated according to the manufacturer’s instructions.

### EdU assay

CA27 and SCC9 cells were inoculated in 12-well plates. After 24 h, we used the BeyoClick™ EdU-594 kit (Beyotime, Shanghai, China, C0078S) according to the manufacturer’s instructions for subsequent experiments. Finally, the results were acquired via fluorescence microscopy.

### Colony formation assay

In each well of a six-well plate, 1,000-well-grown cells were inoculated and cultured for 10 days. The cells were then fixed using paraformaldehyde for 15 min at room temperature, washed twice with PBS, and stained with crystal violet. Finally, the plates were washed with PBS again and photographed under a light microscope.

### Tumorsphere formation assay

Each well of a low-adhesion six-well plate was inoculated with 1,000-well-grown cells. The cells were cultured in standard CSC medium consisting of DMEM-F12 supplemented with 2% B27 (Life Technologies, Waltham, MA), 100 U/ml penicillin, 20 ng/ml human EGF, 100 ng/ml streptomycin (PeproTech), and 10 ng/ml human bFGF (PeproTech). After 10 days, the number of tumorspheres was counted under an inverted microscope.

### Western blot

Well-grown cells were lysed by radioimmunoprecipitation assay (RIPA), and the protein concentration was determined by BCA (Beyotime, P0010). Proteins were separated by SDS‒PAGE and then transferred to a polyvinylidene difluoride (PVDF) membrane. After 1 h of incubation with 5% skim milk at room temperature, the appropriate primary antibody was added, and the samples were incubated at 4 °C overnight. On the following day, subsequent tests were performed using ECL kits, and the results were visualized with an Odyssey System (LI-COR Biosciences) after the addition of the corresponding secondary antibody. EphA2 (#66736-1-Ig, 1:5,000), CD133 (#18470-1-AP, 1:2,000), CD44 (#15675-1-AP, 1:2,000), ALDH1A1 (#15910-1-AP, 1:1,000) and YAP (#13584-1-AP, 1:2,000 and #66900-1-Ig, 1:5,000) antibodies were purchased from Proteintech (Wuhan, China). Antibodies against ERK1/2 (#4695, 1:1,000) and phospho-ERK1/2 (p-ERK) (#4376, 1:1,000) were purchased from Cell Signaling Technology (Danvers, MA, USA). KLF4 (#A13673, 1:500) and TEAD3 (#A7454, 1:500) antibodies were purchased from ABclonal (Wuhan, China).

### Coimmunoprecipitation (Co-IP) assay

The operation was performed following the manufacturer’s instructions for Universal Magnetic co-IP kits purchased from Active Motif (54,002, USA). Western blotting was performed as previously described. Antibodies against YAP (Proteintech, #13584-1-AP) or TEAD3 (ABclonal, #A7454) were used.

### RNA extraction and reverse transcription-quantitative polymerase chain reaction (RT‒qPCR)

Total RNA was obtained from well-grown cells using RNAiso Plus (Takara, Tokyo, Japan), and the concentration of total RNA was measured. Subsequently, cDNA was obtained by reverse transcription of 1 µg of total RNA using a HiScript RT reagent kit (Vazyme Biotech, China). Finally, a Bio-Rad CFX 96TM system was used with ChamQ SYBR qPCR Master Mix (Vazyme Biotech, China) to detect mRNA expression. For primer sequences, see Supplementary Table 3.

### RNA interference and lentiviral transfection

The siRNAs were purchased from Hanbio Technology (Shanghai, China). For sequences of RNA interference, see Supplementary Table 4. The recombinant lentivirus was purchased from GeneChem (Shanghai, China). Lentiviruses named shEphA2 and shKLF4 were used to interfere with the expression of EphA2 and KLF4, respectively. Lentiviruses named OE-YAP and OE-KLF4 were used to increase the expression of YAP and KLF4, respectively. Cells were transfected with lentivirus for 12 h and screened with puromycin (2 µg/ml) for 1 week to establish stably transfected cell lines. pLV3-EphA2, purchased from MiaoLing (Wuhan, China), was used to increase the expression of EphA2.

### Dual luciferase reporter gene assay

The phRL-TK plasmid and pGL3 luciferase plasmids containing the KLF4 promoter were purchased from MiaoLing (Wuhan, China). OE-YAP OSCC cells and control cells were cultured in 24-well plates, and 0.1 µg of the PGL3-basic-H-KLF4 plasmid and 0.02 µg of the phRL-TK plasmid were transfected into each well using Lipofectamine 8,000 (Beyotime, Shanghai, China). Forty-eight hours later, we measured luciferase activity using the Dual Luciferase Reporter Assay Kit (Beyotime, Shanghai, China).

### Chromatin immunoprecipitation (ChIP)-qPCR

The Pierce Magnetic ChIP Kit purchased from Thermo Fisher Scientific Inc. (26,157, USA) was used according to the manufacturer’s instructions. TEAD3 and anti-IgG antibodies were added to the corresponding samples. Subsequently, qPCR was performed as described above. For primer sequences, see Supplementary Table 5.

### Animals

The mouse experiments were approved by the Laboratory Animal Ethics Committee, Hospital of Stomatology, Wuhan University. Female BALB/c nude mice (aged 6 weeks) were purchased from Beijing Vital River Laboratory Animal Technology Co., Ltd. (Beijing, China). The mice were randomly divided into different groups according to the aim of the experiment. For tumour-initiating frequency analysis, 10^3^, 10^4^, and 10^5^ SCC9 cells were resuspended in 100 µl of PBS and injected subcutaneously into the mice. For the tumour growth assay, 3 × 10^6^ SCC9 cells were resuspended in 100 µl of PBS and injected subcutaneously into the mice (*n* = 7/group). No animals were excluded from the analysis. We measured the size of the tumours blindly every 7 days and calculated tumour volumes using the formula (width^2^ × length)/2. Tumour-initiating frequency analysis was performed using Extreme Limiting Dilution Analysis software (http://bioinf.wehi.edu.au/software/elda/index.html) [28].

### Immunohistochemical staining (IHC)

The tissues were fixed in formalin, paraffin-embedded and cut into 5 μm sections. This step was followed by dewaxing, gradient hydration and microwave antigen retrieval. Afterwards, the corresponding primary antibodies against EphA2 (Proteintech, #66736-1-Ig, 1:200), CD44 (Proteintech, #60224-1-Ig, 1:200), YAP (Proteintech, #66900-1-Ig, 1:1,000) and Ki67 (ABclonal, #A20018, 1:200) were added and incubated at 4 °C overnight. The next day, after washing and adding the secondary antibody and streptavidin-peroxidase (UltraSensitiveTM SP kit, Maixin, Fuzhou, China), DAB was used to visualize the results.

### Correlation scatterplot

We used the ggpubr package of R to analyse the GSE181805, GSE6791 and GSE112026 for plotting the correlation scatterplot between YAP and EphA2.

### Statistical analysis

Student’s t test or analysis of variance (ANOVA) was used in the relevant section. Pearson rank correlation was used for the correlation tests. All the results were calculated from at least three independent experiments and are presented as the means ± SDs. *P* < 0.05 was considered to indicate statistical significance (*P < 0.05, **P < 0.01, ***P < 0.001 and ****P < 0.0001).

## Results

### EphA2 is highly expressed in OSCC and is associated with poor prognosis

**Fig. 1:**
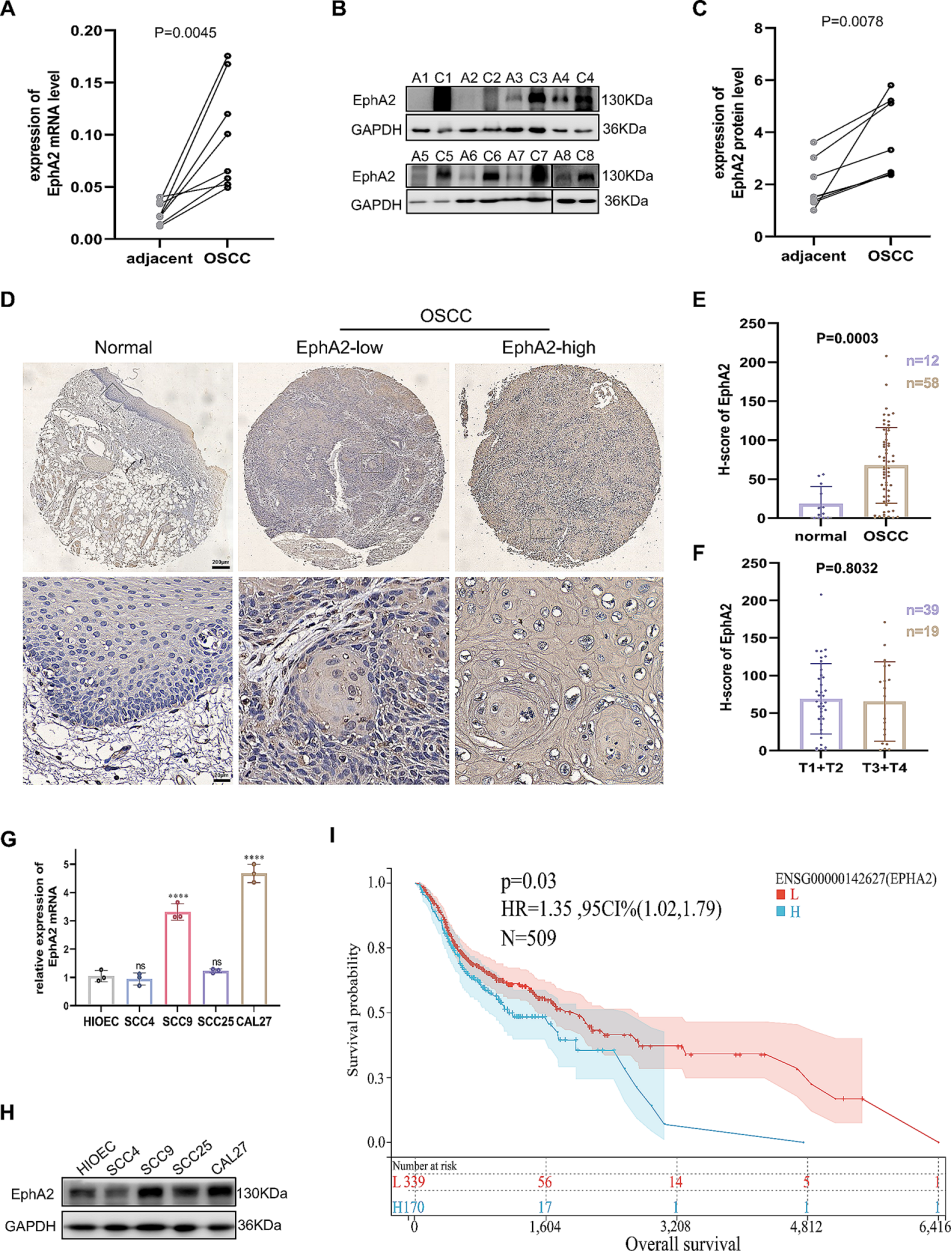
EphA2 is overexpression in OSCC and is contributed to poor prognosis. **A** Ephrin receptors A2 (EphA2) mRNA level was higher in oral squamous cell carcinoma (OSCC) tissue than its paired adjacent non-cancer tissues (*n* = 8). **B, C** EphA2 protein expression was higher in OSCC tissue than its paired adjacent non-cancer tissues (*n* = 8) (**B**), and its quantitative analysis (**C**). **D, E** Representative immunohistochemistry (IHC) images of EphA2 protein in clinical specimens (*n* = 70) (**D**), and its histochemistry score (H-score) (**E**). Scale bars: 200 μm for global picture, and 20 μm for enlarged picture. **F** EphA2 expression was not associated with tumor size of OSCC tissues. **G, H** EphA2 mRNA and protein expression in OSCC cell lines and human immortalized oral epithelial cell (HIOEC). **I** Highly expressed EphA2 mRNA levels was associated with shorter overall survival time in head and neck squamous cell carcinoma created by SangerBox website. *p* = 0.03

As a promising target in cancer, EphA2 has been studied in certain cancers, but research on EphA2 in OSCC is limited [23]. Therefore, we detected its expression in OSCC tissues and paired adjacent noncancer tissues by RT‒qPCR (Fig. 1A) and western blotting (Fig. 1B, C). In addition, a tissue chip comprising 58 OSCC tissues and 12 normal oral mucosa tissues was used for immunohistochemistry (IHC) staining for EphA2 (Fig. 1D, E). The results showed that EphA2 expression was significantly elevated in OSCC tissue compared with that in oral normal tissue but was not correlated with tumour size, lymph node metastasis, distant metastasis, or tumour stage (Fig. 1F, Supplementary Fig. 1A-C). In addition, we found that the mRNA and protein expression of EphA2 in OSCC cell lines (SCC4, SCC9, SCC27, and CAL27) was not lower than that in human immortalized oral epithelial cells (HIOECs). SCC9 and CAL27 cells had increased EphA2 mRNA and protein expression (Fig. 1G, H). Next, we explored the relationship between EphA2 and the overall survival rate of OSCC patients via the SangerBox website (http://www.sangerbox.com/) and found that higher EphA2 expression was associated with shorter survival (*P* = 0.03) (Fig. 1I), which was consistent with our previous study [27]. Taken together, the above results indicate that EphA2 might be a cancer-promoting factor in OSCC.

### EphA2 promotes the CSC properties of OSCC cells

**Fig. 2:**
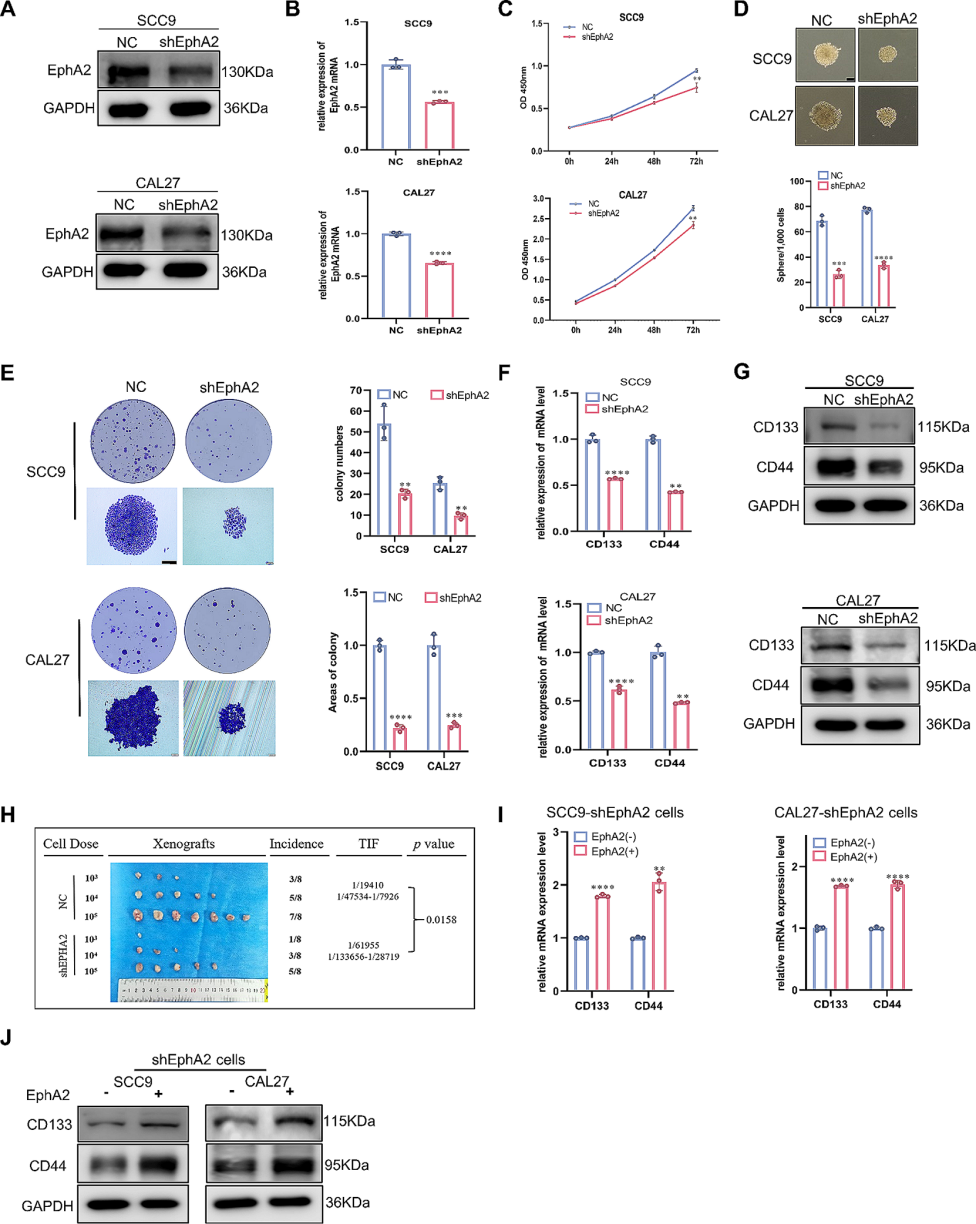
EphA2 promotes the stemness of OSCC cells. **A, B** EphA2 protein (**A**) and mRNA (**B**) expression in OSCC cells transduced with EphA2 shRNAs. **C-E** Knockdown of EphA2 impaired the proliferation (**C**), sphere formation (**D**) (scale bars: 100 μm), and colony formation ability of OSCC cells (**E**) (scale bars: 200 μm). **F, G** Knockdown of EphA2 reduced the mRNA (**F**) and protein (**G**) expression of cancer stem cells (CSC)-related markers. **H** EphA2 knockdown cells were injected subcutaneously in the BALB/c nude mice. The tumor-initiating frequency was determined by ELDA software [28]. **I, J** Overexpression of EphA2 enhanced the mRNA (**I**) and protein (**J**) expression of CSC-related markers in shEphA2-OSCC cells

CSC determine the occurrence and development of many cancers, including OSCC [29–31]. EphA2, a cancer-promoting factor in OSCC, might be involved in regulating CSC properties. To verify this hypothesis, the EphA2 knockdown SCC9 and CAL27 cell lines were constructed with shRNAs (Fig. 2A, B). We selected SCC9 and Cal27 cells because they exhibited increased EphA2 expression among OSCC cells (Fig. 1G, H). We found that the proliferation, sphere-forming, and colony-forming abilities of the EphA2 knockdown cells were impaired (Fig. 2C-E). In addition, drug tolerance is a property of CSCs associated with multiple mechanisms, including the expression of ATP-binding cassette transporters, escape from apoptosis and the expression of some noncoding RNAs [32]. Indeed, sensitivity to cisplatin was increased in EphA2 knockdown cells (Supplementary Fig. 2A). In addition, EphA2 knockdown reduced the mRNA and protein expression of the CSC-related markers CD44 and CD133 (Fig. 2F, G). In addition, EphA2-knockdown cells exhibited a decreased tumour-initiating frequency and tumour volume in vivo (Fig. 2H). Then, we overexpressed EphA2 in the shEphA2 cell line (Supplementary Fig. 2B, C) and found that the inhibition of proliferation and sphere formation and decreased expression of CSC-related markers were reversed (Supplementary Fig. 2D, E; Fig. 2I, J). Collectively, these results reveal that EphA2 can enhance the CSC properties of OSCC cells.

### KLF4 mediates EphA2-enhanced CSC properties in OSCC

**Fig. 3:**
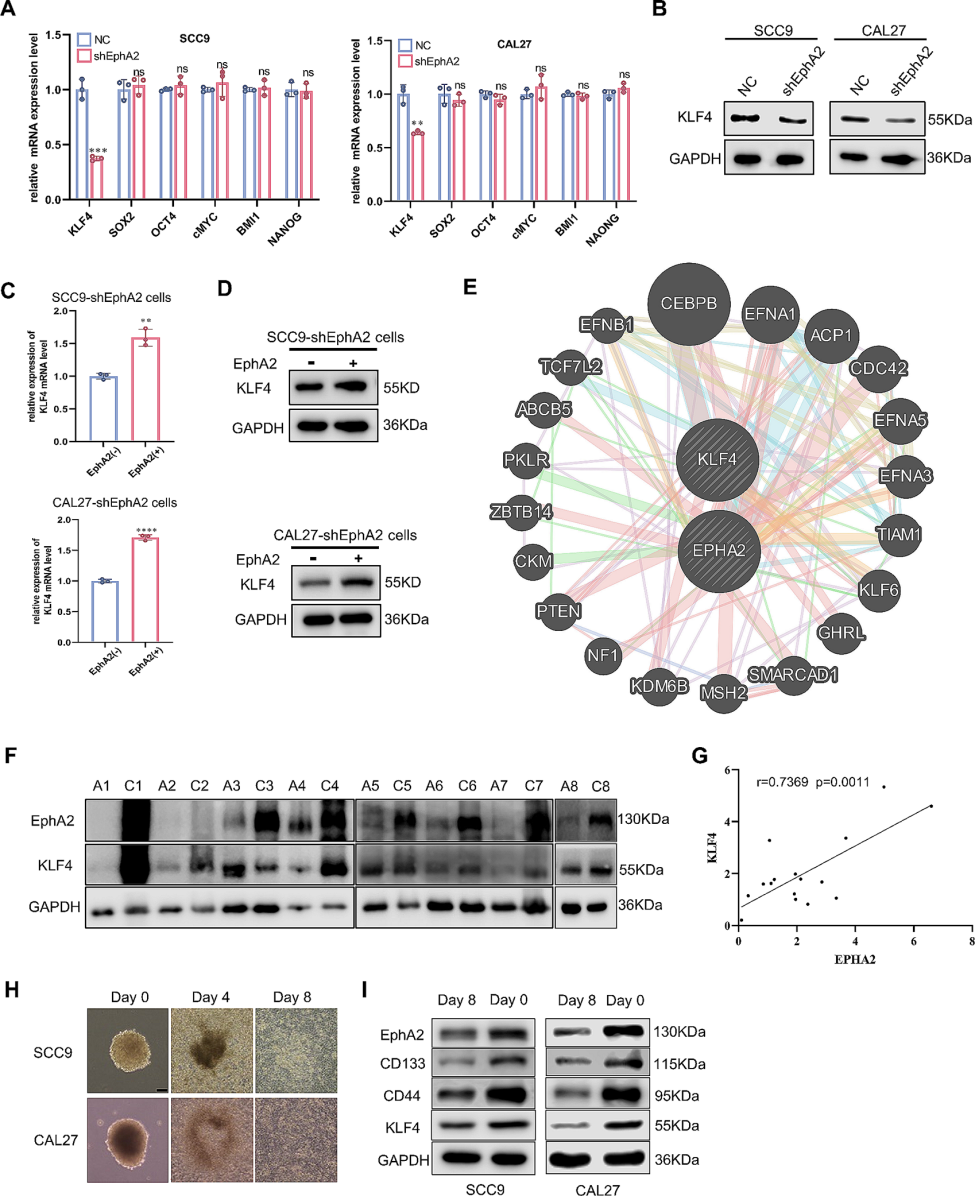
KLF4 is a downstream target of EphA2 in OSCC. **A** The mRNA expression of CSC-related transcription factor in shEphA2-OSCC cells. **B** Knockdown of EphA2 reduced the Krüppel-like factor 4 (KLF4) protein expression in OSCC cells. **C, D** Overexpression of EphA2 enhanced the KLF4 mRNA (**C**) and protein (**D**) expression in shEphA2-OSCC cells. **E** Some co-regulated proteins of EphA2 and KLF4 acquired from geneMANIA [37]. **F, G** The EphA2 and KLF4 protein expression in OSCC tissues and paired adjacent non-cancer tissues (*n* = 8) (**F**), and the correlation coefficient between EphA2 and KLF4 (**G**), which was created by statistical analysis of the protein expression in F. **H, I** Tumor sphere was induced to differentiation into monolayer by serum for 8 days. Representative images were shown (H), and the protein expression of KLF4, EphA2 and CSC-related markers in monolayer cells (day 8) was lower in than tumor sphere (day 0) (**I**). Scale bar: 100 μm

To elucidate the mechanism by which EphA2 promotes the CSC properties of OSCC, we focused on its relationship with several TFs that are extremely important for the maintenance of CSC [33]. The RT‒qPCR results showed that the mRNA expression of KLF4 was dramatically reduced after EphA2 knockdown, while that of SOX2, OCT4, c-MYC, BMI1, and NANOG was unchanged (Fig. 3A), and the western blotting results were similar (Fig. 3B). KLF4 is necessary for the formation of induced pluripotent stem cells, and its role as an important TF in CSC is gradually becoming known [34–36]. Accordingly, the mRNA and protein levels of KLF4 were increased when EphA2 was overexpressed (Fig. 3C, D). The results acquired from GeneMANIA [37] showed that EphA2 and KLF4 had numerous coregulated proteins (Fig. 3E). In addition, western blotting was performed and revealed that KLF4 expression was positively correlated with EphA2 expression in 8 pairs of fresh clinical tissues (Fig. 3F, G). Moreover, the protein expression in the monolayer, which was generated by serum induction of tumorspheres for 8 days, and protein expression in the tumorsphere was detected using western blotting. The results showed that EphA2 and KLF4 were highly coexpressed in the tumorsphere, and the levels of CD44 and CD133 were greater than those in the monolayer (Fig. 3H, I). In summary, these findings suggest that KLF4 is a downstream target of EphA2 in promoting the CSC properties of OSCC.

**Fig. 4:**
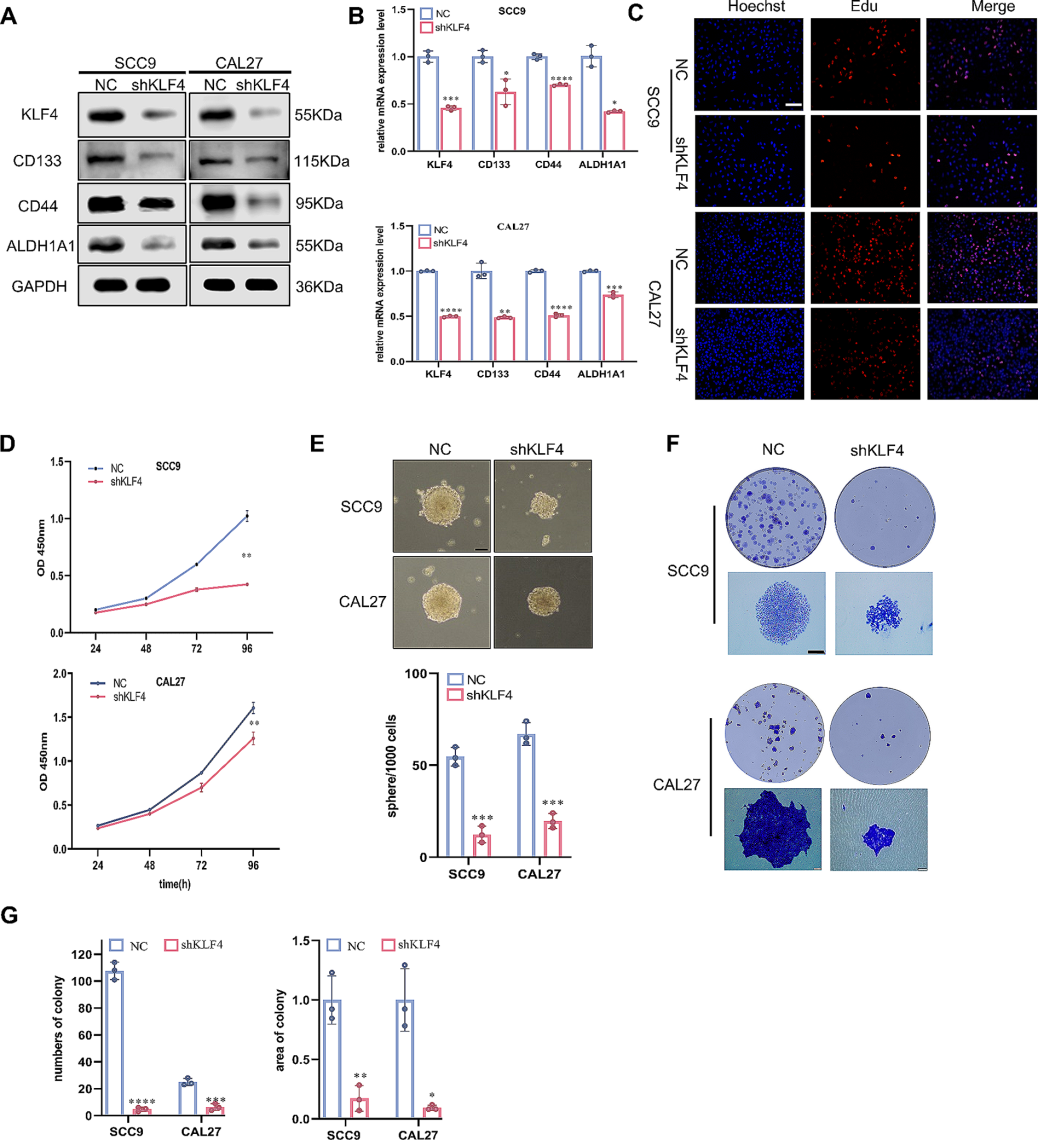
KLF4 promotes the stemness of OSCC cells. **A, B** Knockdown of KLF4 reduced the protein (**A**) and mRNA (**B**) expression of CSC-related markers in OSCC cells. **C, D** Knockdown of KLF4 impaired the proliferation of OSCC cells evaluate by Edu (**C**) and cell counting cit-8 assay (**D**). Scale bars:100 μm. **E-G** Knockdown of KLF4 impaired the sphere formation (**E**) and colony formation ability (**F, G**). Scale bars: 100 μm in E and 200 μm in F

Having confirmed that KLF4 is a downstream target of EphA2, the next question is whether KLF4 can promote the CSC properties of OSCC. As shown in Supplementary Fig. 3A, KLF4 was positively correlated with cancer stemness in a variety of cancers, including HNSCC. Correspondingly, knockdown of KLF4 reduced the protein and mRNA expression of the CSC-related markers CD44, CD133, and ALDH1A1 in OSCC cells (Fig. 4A, B). Furthermore, the EdU and CCK-8 assays demonstrated a decrease in OSCC cell proliferation following KLF4 knockdown (Fig. 4C, D). Moreover, sphere-forming and colony-forming abilities were impaired, while cisplatin sensitivity was enhanced when KLF4 was suppressed (Fig. 4E-G, Supplementary Fig. 3B). These findings suggest that KLF4 promotes CSC properties in OSCC.

The above results indicate that EphA2 enhances the stemness of OSCC cells through KLF4.

### EphA2 enhances the nuclear translocation of YAP by activating the ERK pathway

**Fig. 5:**
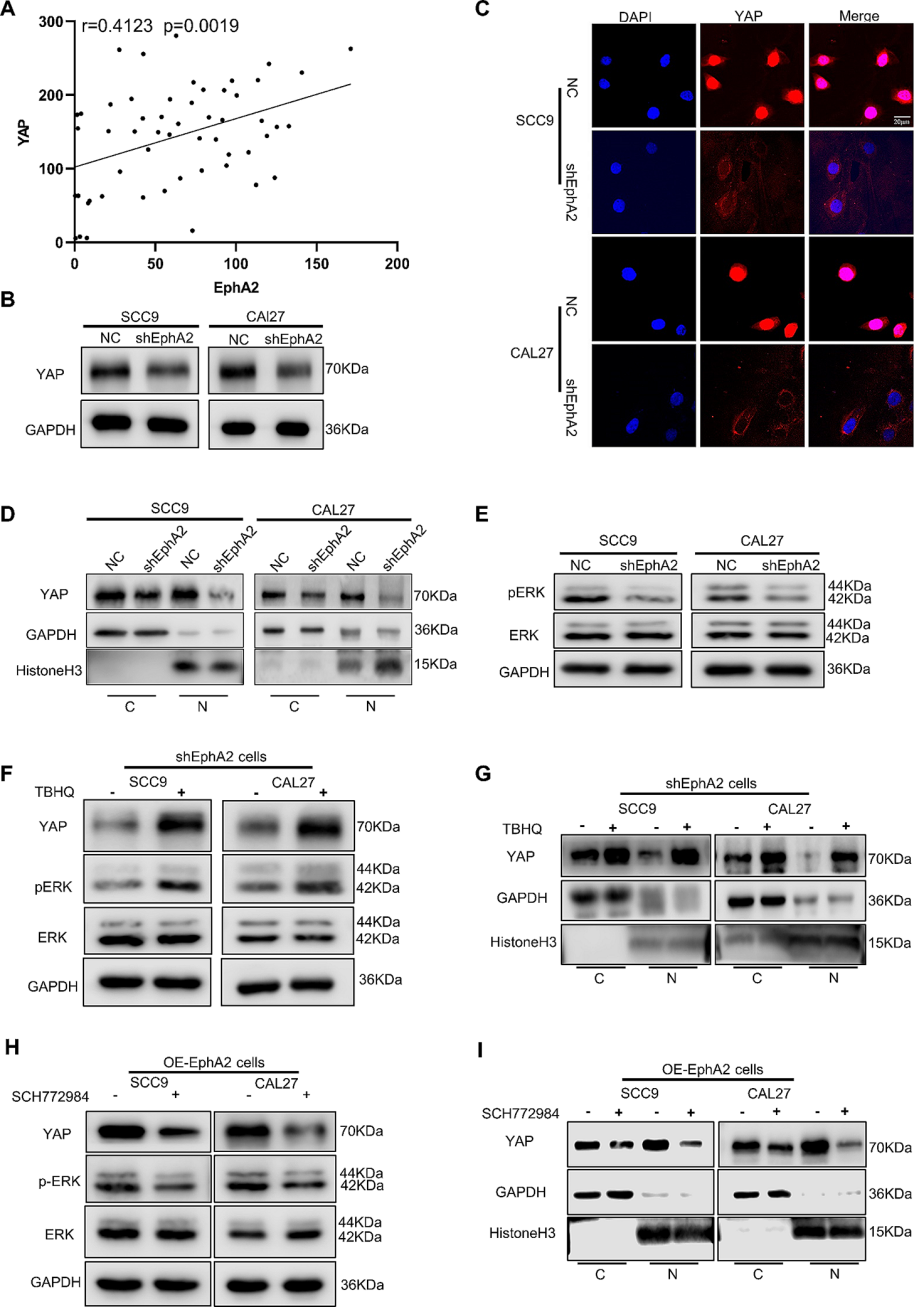
EphA2 promotes the nuclear translocation of YAP by activating ERK pathway. **A** The correlation between YAP and EphA2 acquired by IHC. **B** Knockdown of EphA2 reduced the expression of YAP. **C, D** Knockdown of EphA2 reduced the nuclear expression of YAP detected by confocal micrographs (**C**) and western blotting (**D**). Scale bars: 20 μm. **E** Knockdown of EphA2 reduced the pERK protein expression. **F, G** Total (**F**) and nuclear (**G**) expression of YAP were upregulated after adding ERK activator (TBHQ, 5 µM) in shEphA2-OSCC cells. **H, I** Total (**H**) and nuclear (**I**) expression of YAP were decreased after adding ERK inhibitor (SCH772984, 10 µM) in OE-EphA2-OSCC cells

As KLF4 plays an important role in EphA2-mediated stemness of OSCC, it is necessary to investigate how EphA2 regulates KLF4. Several studies have shown that YAP, an effector of the Hippo pathway, is the main mediator of RTK signalling [38, 39] and is strongly associated with CSC properties in many cancers [40–42]. Indeed, using public datasets (Supplementary Fig. 4A) and our tissue microarrays (Fig. 5A), we found that YAP expression was positively correlated with EphA2 expression, and that EphA2 knockdown reduced YAP protein expression (Fig. 5B). The nucleus is where YAP, a transcriptional coactivator, performs its physiological functions [43]. Therefore, we hypothesized that EphA2 also affects the intracellular localization of YAP. Indeed, knockdown of EphA2 reduced the expression of YAP in the nucleus, as detected by confocal microscopy and western blotting (Fig. 5C, D). These results demonstrate that EphA2 regulates the function of YAP. However, the specific mechanism is unknown. Since EphA2 is a membrane protein and YAP functions in the nucleus, there should be a mediator between these two proteins.

The ERK pathway is one of the main downstream pathways of the RTK family, which includes EphA2 [39, 44–46]. Interestingly, ERK and YAP are closely related in many cancers, including HNSCC [39, 47]. In addition, ERK can enhance the transcription of *YAP* by inducing the degradation of the transcriptional repressor Capicua in cancer cells [48]. Moreover, YAP nuclear translocation can also be induced by phosphorylated ERK in hepatocellular tumour cells [49]. To determine whether the ERK pathway is a mediator between EphA2 and YAP, several experiments were performed. Western blotting analysis revealed that the ERK pathway was impaired when EphA2 was knocked down in OSCC cells (Fig. 5E). Correspondingly, total YAP expression was upregulated when the ERK pathway was activated by an ERK activator (TBHQ, 5 µM) (Fig. 5F), and more YAP was detected in the nucleus (Fig. 5G, Supplementary Fig. 4B). To further confirm the above results, we generated EphA2-overexpressing cells (Supplementary Fig. 4C). As shown, the total amount of YAP and the amount of YAP expressed in the nucleus were reduced in the OE-EphA2 cells treated with the ERK inhibitor (SCH772984, 10 µM) (Fig. 5H, I; Supplementary Fig. 4D). Together, these results prove that EphA2 can promote the nuclear translocation of YAP by activating the ERK pathway.

### The YAP-TEAD3 complex directly activates *KLF4* transcription

**Fig. 6:**
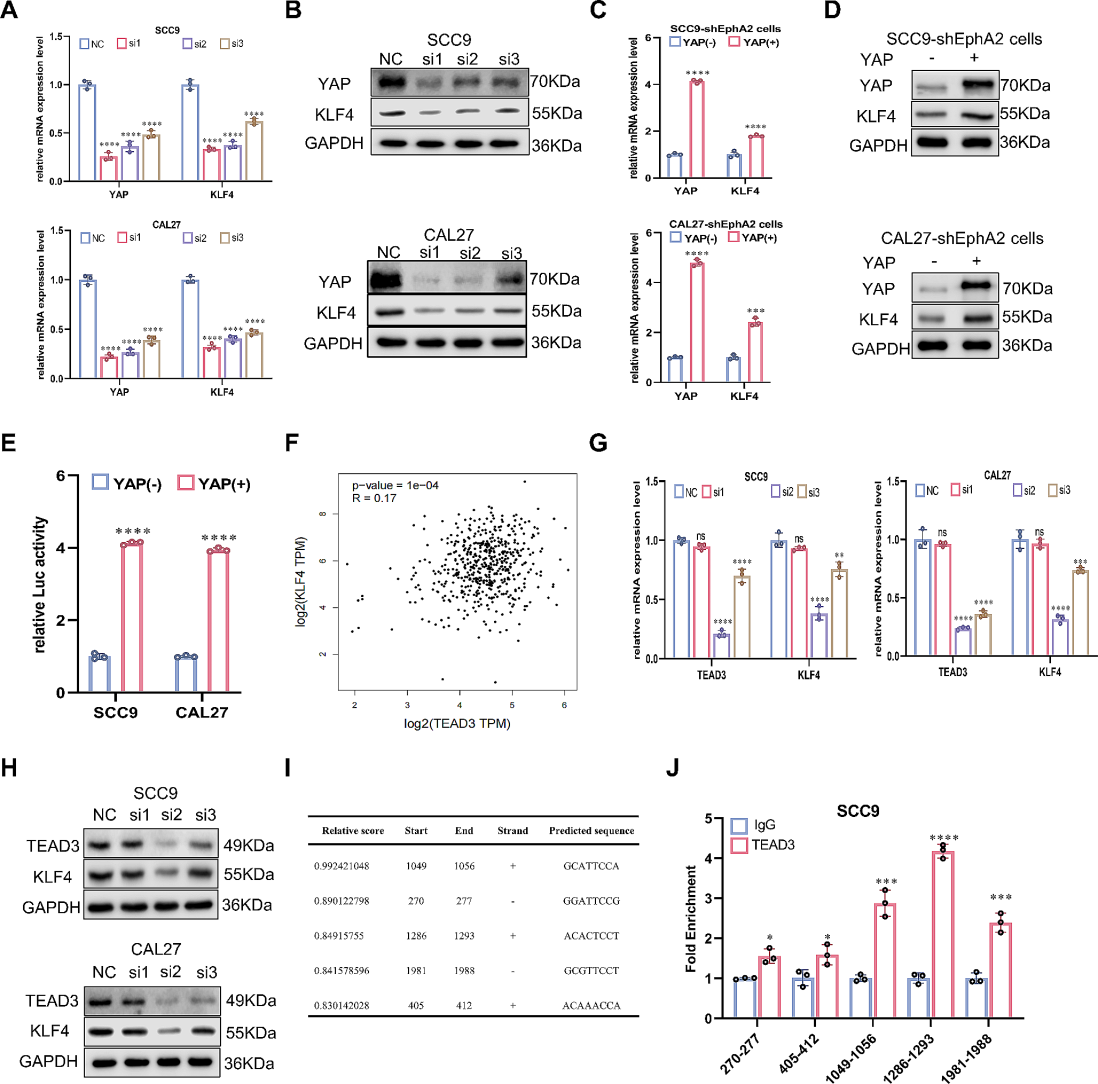
YAP-TEAD3 complex directly activates *KLF4* transcription in OSCC cells. **A, B** Knockdown of YAP by siRNAs reduced the mRNA (**A**) and protein (**B**) expression of KLF4. **C, D** Overexpression of YAP increased the mRNA (**C**) and protein (**D**) expression of KLF4 in shEphA2-OSCC cells. **E** Overexpression of YAP enhanced the transcription activity of *KLF4* detected by dual luciferase reporter gene assay. **F** The correlation between TEAD3 and KLF4 acquired from GEPIA2 [52]. **G, H** Knockdown of TEAD3 by siRNAs reduced the mRNA (**G**) and protein (**H**) expression of KLF4. **I** The potential binding sites of TEAD3 in *KLF4* promoter acquired from JASPAR [53]. **J** The fold enrichment of different binding sits on the promoter of *KLF4* detected by ChIP-qPCR

Given that the connection between EphA2 and YAP has been demonstrated, the next step is to determine whether YAP can regulate KLF4. Knockdown of YAP by siRNAs reduced the mRNA and protein expression of KLF4 (Fig. 6A, B). Knockdown of YAP in OE-EphA2 cells produced similar results (Supplementary Fig. 5A, B), while overexpression of YAP in shEphA2 cells resulted in elevated KLF4 (Fig. 6C, D). Because YAP functions as a transcriptional coactivator and because the above results suggested that YAP enhanced the expression of KLF4, we used a dual luciferase reporter gene assay to determine whether YAP could promote the transcription of *KLF4*. We constructed OE-YAP OSCC cells (Supplementary Fig. 5C) and then transferred plasmids containing the KLF4 promoter into them. As shown in Fig. 6E, YAP significantly increased the transcriptional activity of *KLF4*.

To promote transcription, YAP needs to bind to its target genes. However, YAP lacks a DNA binding domain and therefore needs to interact with other transcription factors that have DNA binding domains, which is the reason why YAP is regarded as a transcription coactivator. TEADs are well known to cooperate to mediate the transcription of YAP target genes [50, 51]. Consequently, YAP may regulate the expression of *KLF4* by forming a complex with TEADs. There are four types of TEADs, TEAD1-4, and we screened the member that was most strongly correlated with KLF4 expression via GEPIA2 [52]. As shown in Supplementary Fig. 5D and Fig. 6F, TEAD3 had the greatest effect. In addition, the coimmunoprecipitation results confirmed that YAP could interact with TEAD3 (Supplementary Fig. 5E). The above results suggested that TEAD3 might act as a partner of YAP to regulate *KLF4* expression. To confirm this, OSCC cells were transfected with siRNA targeting TEAD3, and we found that TEAD3 knockdown also reduced the mRNA and protein expression of KLF4 (Fig. 6G, H). To further explore the binding site between TEAD3 and *KLF4*, JASPAR [53] was used, and we predicted five potential binding sites for TEAD3 in the promoter region of *KLF4* (Fig. 6I). Subsequently, a ChIP‒qPCR assay was performed. The results showed that the region from 1286 to 1293 was the most likely binding site (Fig. 6J). Overall, the above results suggest that the YAP-TEAD3 complex can enhance the transcriptional activity of *KLF4*.

### Knockdown of EphA2 inhibits tumour growth and impairs the stemness of OSCC cells in vivo

**Fig. 7:**
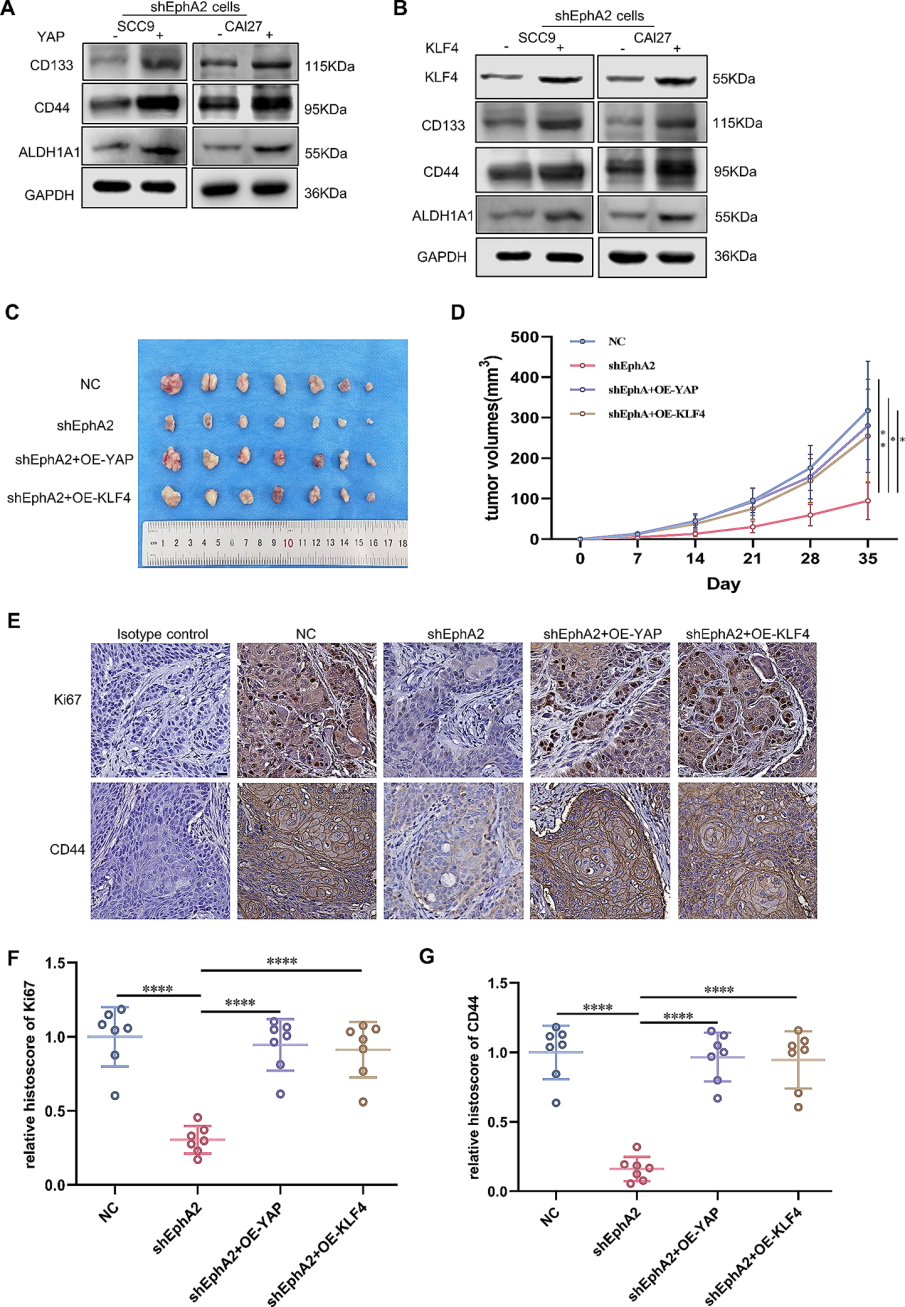
EphA2 inhibits the growth and stemness of OSCC cells in vivo. **A, B** The suppression of CSC-related markers expression by knockdown of EphA2 could be reversed by overexpression of YAP (**A**) or KLF4 (**B**). **C** Tumor images of cells pretreated with knockdown of EphA2 or overexpression of YAP or overexpression of KLF4 (cell dose = 3 × 10^6^, *n* = 7). **D** Growth curves of tumors that measured every 7 days. **E-G** Representative IHC images of Ki67 and CD44 in excised tumor tissues (**E**), and their histochemistry score (**F, G**). Scale bars: 20 μm

Since we revealed the mechanism by which EphA2 promotes the stemness of OSCC cells in vitro, whether EphA2 plays a similar role in vivo remains to be investigated. We established four cell lines, NC, shEphA2, shEphA2 + OE-YAP and shEphA2 + OE-KLF4, in vitro and then examined their effects on sphere-forming, and colony-forming abilities and CSC-related markers expression (Supplementary Fig. 6.A-D; Fig. 7A, B) The results showed that YAP and KLF4 could reverse the effects of EphA2 knockdown. Subsequently, using a subcutaneous xenograft mouse tumour model established by injecting SCC9 cells divided into NC, shEphA2, shEphA2 + OE-YAP, and shEphA2 + OE-KLF4 groups, we found that EphA2 knockdown reduced the tumour volume (Fig. 7C, D) and weight (Supplementary Fig. 6E), while YAP or KLF4 overexpression reversed these effects. In addition, immunohistochemical staining revealed that the lowest expression of CD44 and Ki67 was detected in the shEphA2 group, which was also reversed in the shEphA2 + OE-YAP and shEphA2 + OE-KLF4 groups (Fig. 7E-G). These results were consistent with in vitro experiments. Collectively, these results suggest that EphA2 knockdown inhibits OSCC growth and suppresses OSCC stemness in vivo.

## Discussion

**Fig. 8:**
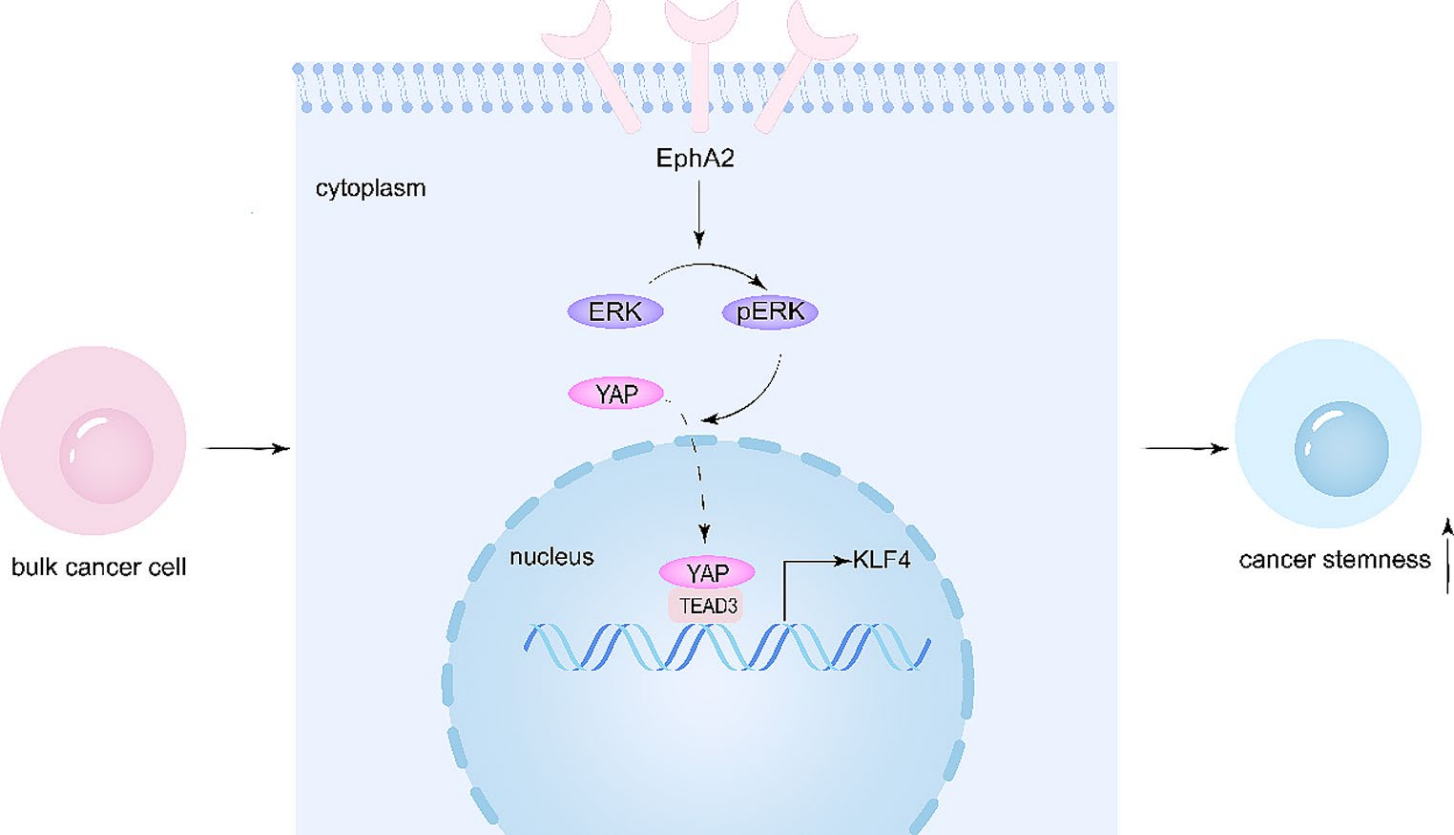
A schematic model of this study. EphA2 activates the ERK pathway, which leads to nuclear translocation of YAP. YAP subsequently binds to TEAD3 to form a transcriptional complex, which activates the transcription of *KLF4*, a key transcription factor for CSCs. This ultimately enhances OSCC cell stemness

Cancer stem cells are key players in the development, progression and treatment resistance of OSCC [54–56], and finding effective targets to eradicate CSCs is a pressing issue. In this study, we found that EphA2 was highly expressed in patients with OSCC and was related to a worse prognosis. Further studies showed that EphA2 promoted OSCC proliferation, sphere formation, colony formation, and drug resistance, which are associated with CSC properties. Mechanistically, EphA2 promoted YAP nuclear translocation through activation of the ERK signalling pathway, which ultimately led to the upregulation of the CSC-associated transcription factor KLF4. Overall, our study identified the intrinsic mechanism by which EphA2 regulates the stemness of OSCC cells and suggested that EphA2 is a potential target for the treatment of OSCC (Fig. 8).

EphA2, which belongs to the RTK family, is overexpressed in a variety of cancers, including lung cancer, breast cancer, and colorectal cancer [23]. Correspondingly, we found that EphA2 mRNA and protein levels were greater in OSCC tissues than in normal tissues and that a higher level of EphA2 expression was associated with a worse prognosis. This undesirable result may be because EphA2 promotes cancer development and progression in multiple ways, such as by promoting cancer cell proliferation, angiogenesis, and metastasis and facilitating the survival of CSCs [24].

CSCs, a class of cells with self-renewal ability and multidifferentiation potential, are responsible for cancer treatment resistance, recurrence, and metastasis, and as such, the eradication of CSCs is a highly promising therapeutic modality [29, 57]. For example, disrupting the binding of KK-LC-1 to FAT1 can lead to a reduction in ALDH1A1 transcription, which reduces ALDH + cell stemness and viability and ultimately leads to the inhibition of TNCB tumour growth [58]. Interestingly, EphA2 is capable of maintaining stemness not only in breast cancer cells but also in cancers such as hepatocellular carcinoma and colorectal cancer, but its relationship with OSCC stemness has rarely been discussed [26, 59, 60]. In our study, we found that EphA2 promoted CSC-associated phenotypes, such as proliferation, sphere formation, colony formation, drug resistance, and tumour initiation, in OSCC cells, suggesting that EphA2 is necessary for OSCC cells to maintain stemness.

KLF4, a Yamanaka factor, plays a key role in sustaining the stemness of embryonic cells and in the formation of induced pluripotent stem cells [19]. Interestingly, KLF4 is also strongly associated with cancer stemness; for example, KLF4 promotes the formation of spheres, the transcription of stemness-associated genes, and chemoresistance in human osteosarcoma cells [22]. Recent studies have demonstrated that KLF4 has an oncogenic role in OSCC [61, 62]. However, it is unclear whether this role is linked to its ability to promote stemness in OSCC cells. In our study, we found that KLF4 expression was regulated by EphA2, and further studies showed that KLF4 knockdown reduced the expression of CSC-associated markers, inhibited sphere formation, and impaired colony formation in OSCC cells, indicating that KLF4 is involved in regulating OSCC stemness.

YAP, a downstream effector of the Hippo pathway, promotes CSC properties in a wide range of cancers when it enters the nucleus to act as a transcriptional coactivator [63, 64]. Notably, since YAP lacks a DNA-binding domain, other transcription factors need to be involved to function properly, and among these transcription factors, the TEAD family is the most closely related [65–67]. In malignancies, many signalling pathways intersect with the Hippo pathway, of which the RTK/ERK signalling pathway is a prominent pathway [38, 39, 68]. Given that EphA2 belongs to the RTK family, EphA2 may play a similar role. Indeed, our study showed that EphA2 promoted YAP entry into the nucleus, and further study showed that this effect was caused by activation of the ERK pathway. Although the relationship between EphA2 and YAP is supported by our and others’ results, knowledge of the connection between YAP and KLF4 is limited in cancer [69]. In our work, we found that YAP formed a transcriptional complex with TEAD3 to initiate *KLF4* transcription, and inhibition of either YAP or TEAD3 resulted in decreased KLF4 expression.

In summary, our study revealed that EphA2 can regulate the stemness of OSCC cells via the ERK/YAP/KLF4 pathway and that targeting EphA2 may be a prospective method for treating OSCC by eradicating CSCs.

### Electronic supplementary material

Below is the link to the electronic supplementary material.

Supplementary Material 1

## Data Availability

All data generated or analyzed in the current study are available from the corresponding author on reasonable request.

## Abbreviations

CCK-: 8 Cell Counting Kit-8
Co-: IP Coimmunoprecipitation
ChIP: Chromatin immunoprecipitation
CSC: Cancer stem cells
EphA2: Ephrin receptors A2
HIOEC: Human immortalized oral epithelial cells
IHC: Immunohistochemistry
KLF4: Krüppel-like factor 4
OSCC: Oral squamous cell carcinoma
RTK: Receptor tyrosine kinases
RT-qPCR: Reverse transcription-quantitative polymerase chain reaction
TEAD: TEA domain family protein
TFs: Transcription factors
YAP: Yes-associated protein
