# Supplementary material for: EphA2 promotes the transcription of KLF4 to facilitate stemness in oral squamous cell carcinoma
Source: Cell Mol Life Sci. 2024 Jun 25;81(1):278. doi: 10.1007/s00018-024-05325-w (PMC11335203; doi:10.1007/s00018-024-05325-w)
Supplement: Supplementary file 1 — Supplementary Material 1 [file 18_2024_5325_MOESM1_ESM.pdf]

Supplementary Fig. 1

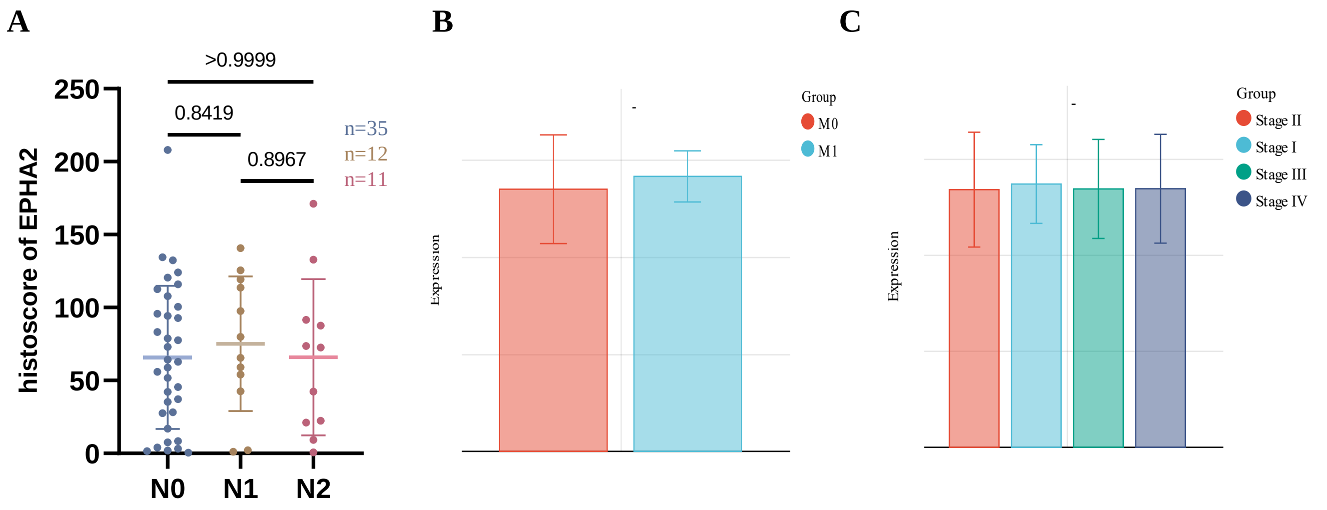

Supplementary Fig. 1 A EphA2 was not associated with OSCC lymph node metastasis acquired from our tissue microarrays. Statistical analyses: one-way ANOVA. Data are presented as means ± SDs. **B,C** EphA2 was not associated with HNSCC distant metastasis (B) and stage (C) acquired from SangerBox.

Supplementary Fig. 2

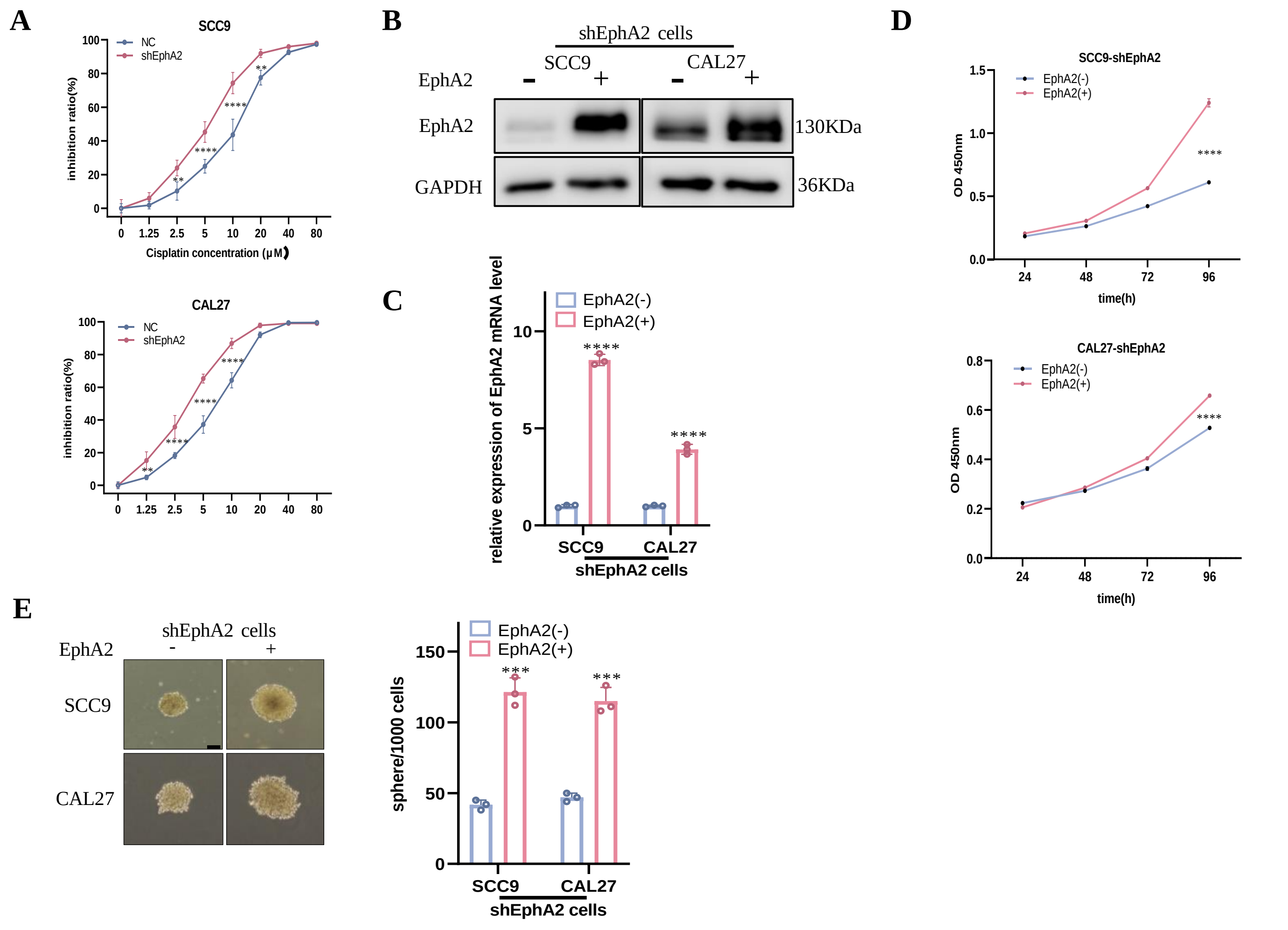

Supplementary Fig. 2. **A** sensitivity to cisplatin was increased in EphA2 knockdown cells. **B, C** The protein (B) and mRNA (C) expression of overexpression EphA2 in shEphA2-OSCC cells. **D** Overexpression of EphA2 enhanced the proliferation ability of OSCC cells. **E** Overexpression of EphA2 enhanced the sphere formation ability of OSCC cells. Scale bars: 100μm.

Supplementary Fig. 3

A

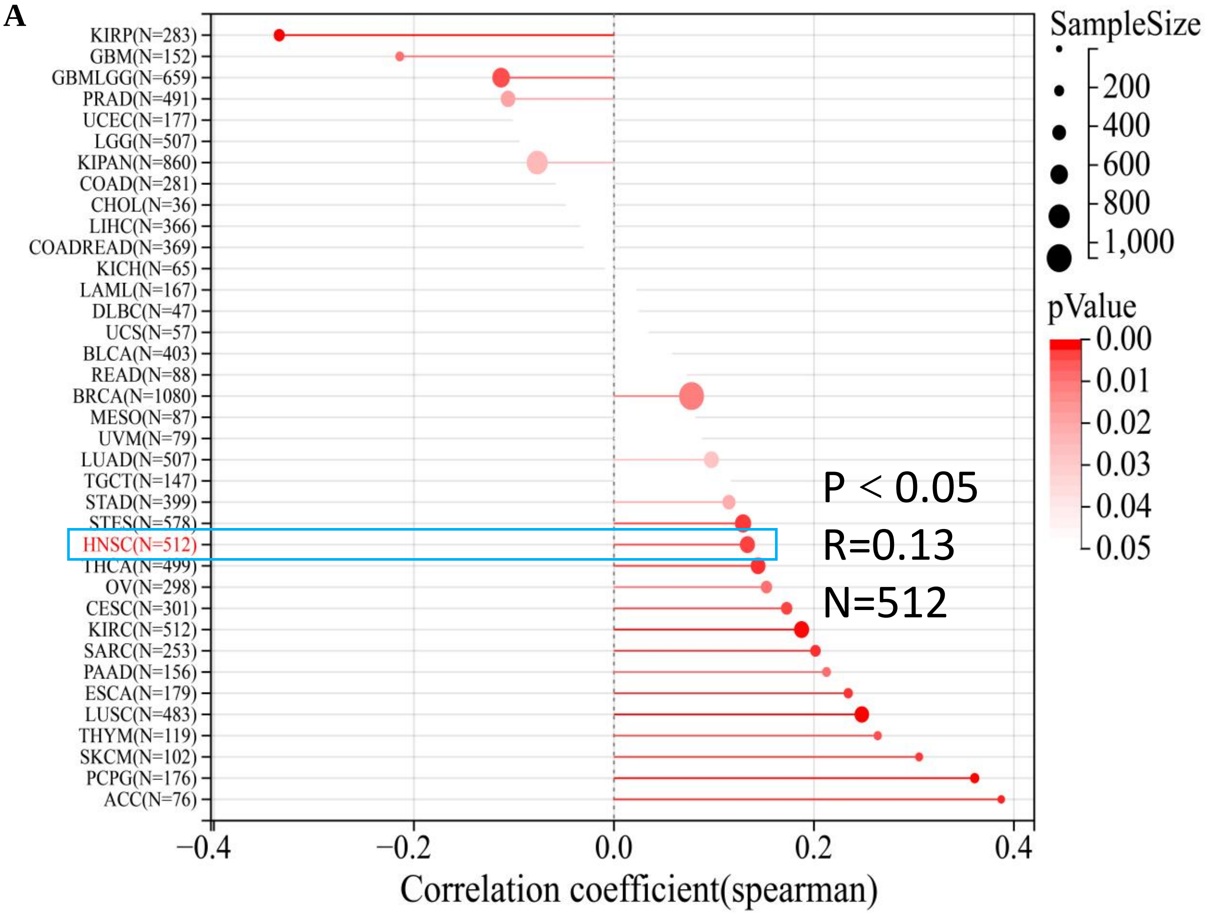

B

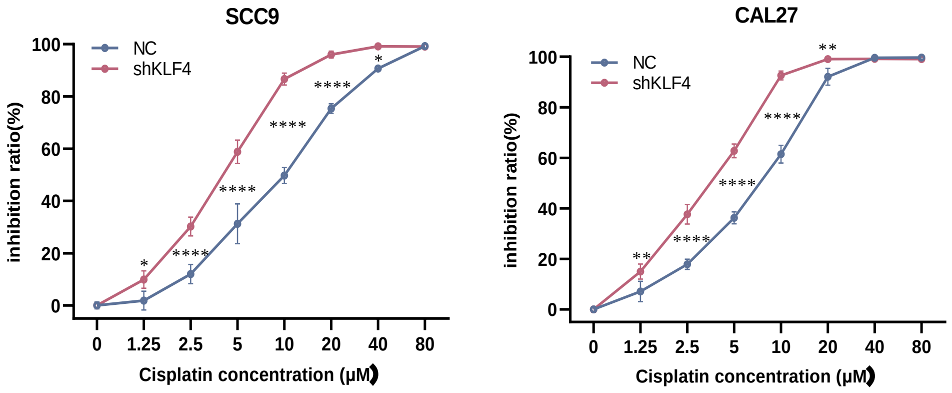

Supplementary Fig. 3. **A** The correlation between KLF4 and stemness scores in head and neck squamous cell carcinoma acquired from SangerBox website. **B** Sensitivity to cisplatin was increased in KLF4 knockdown cells.

Supplementary Fig. 4

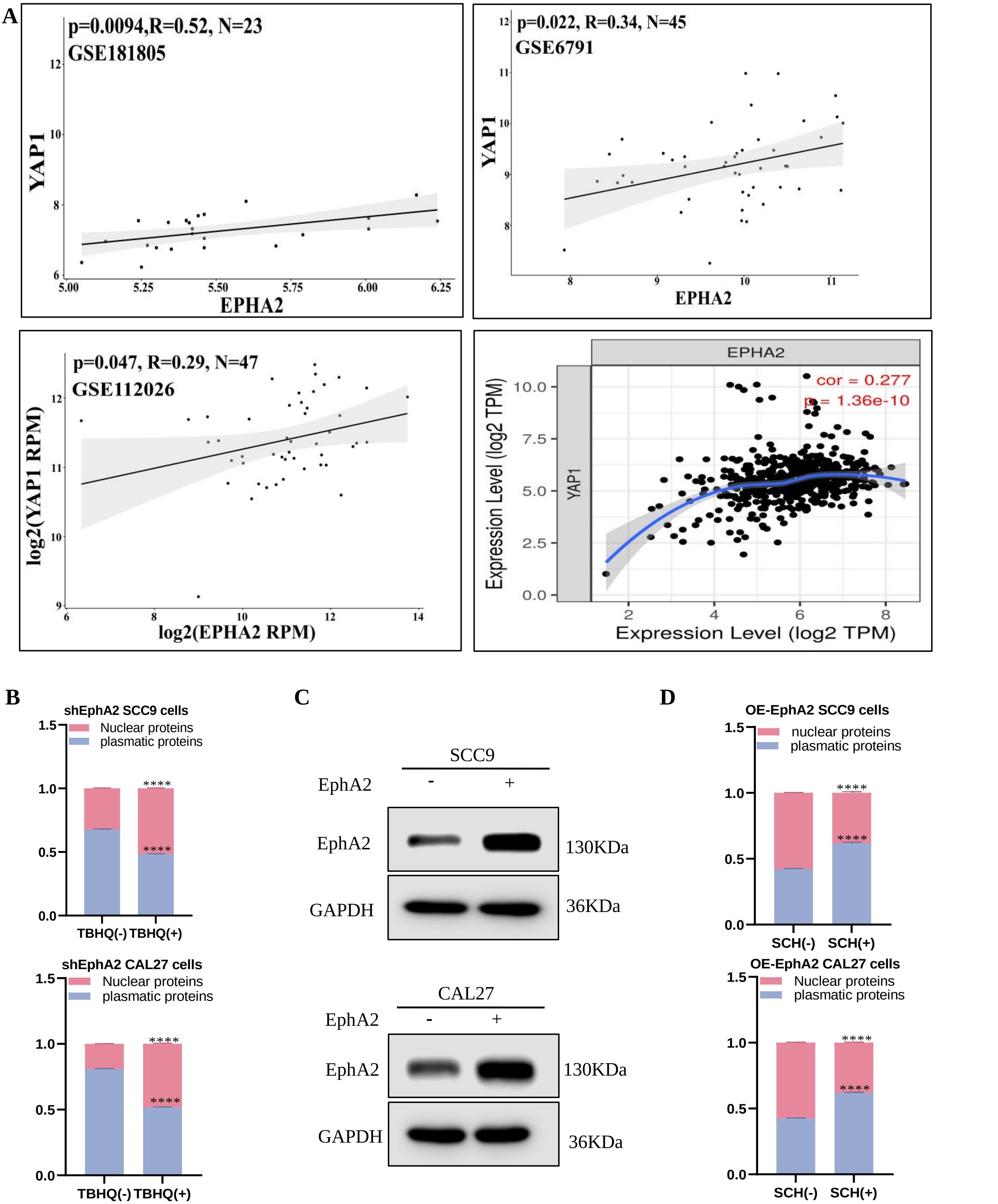

Supplementary Fig. 4 A Different datasets show positive correlation between YAP and EphA2. From left to right, GSE181805 (r=0.52, p=0.0094), GSE6791 (r=0.34, p=0.022), GSE112026 (r=0.29, p=0.047) and the web tool of TIMER (https://cistrome.shinyapps.io/timer/). **B** The percentage of YAP in the nucleus increased after the use of ERK agonists (TBHQ, 5 μM). **C** The effect of EphA2 overexpression. **D** Decreased YAP occupancy in the nucleus following the use of ERK inhibitors (SCH772984 (SCH), 10 μM).

Supplementary Fig. 5

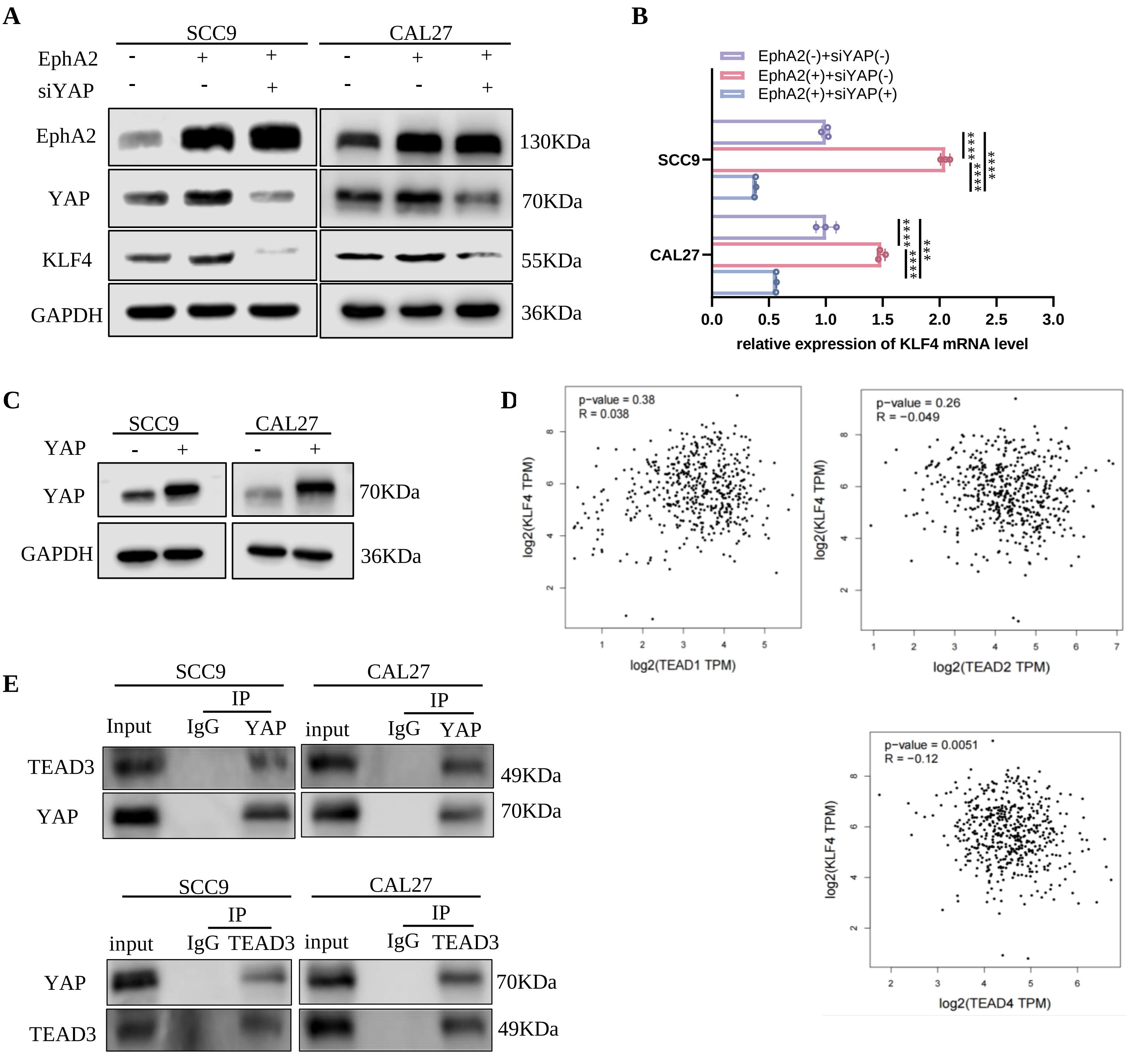

Supplementary Fig. 5. A, B Knockdown of YAP reverses the promotion of KLF4 by OE-EphA2. **C** The effect of YAP overexpression. **D** The correlation among TEAD1, TEAD2 and TEAD4 with KLF4, which was acquired from GEPIA2. **E** The result of coimmunoprecipitation between YAP and TEAD3 in OSCC cells.

Supplementary Fig. 6

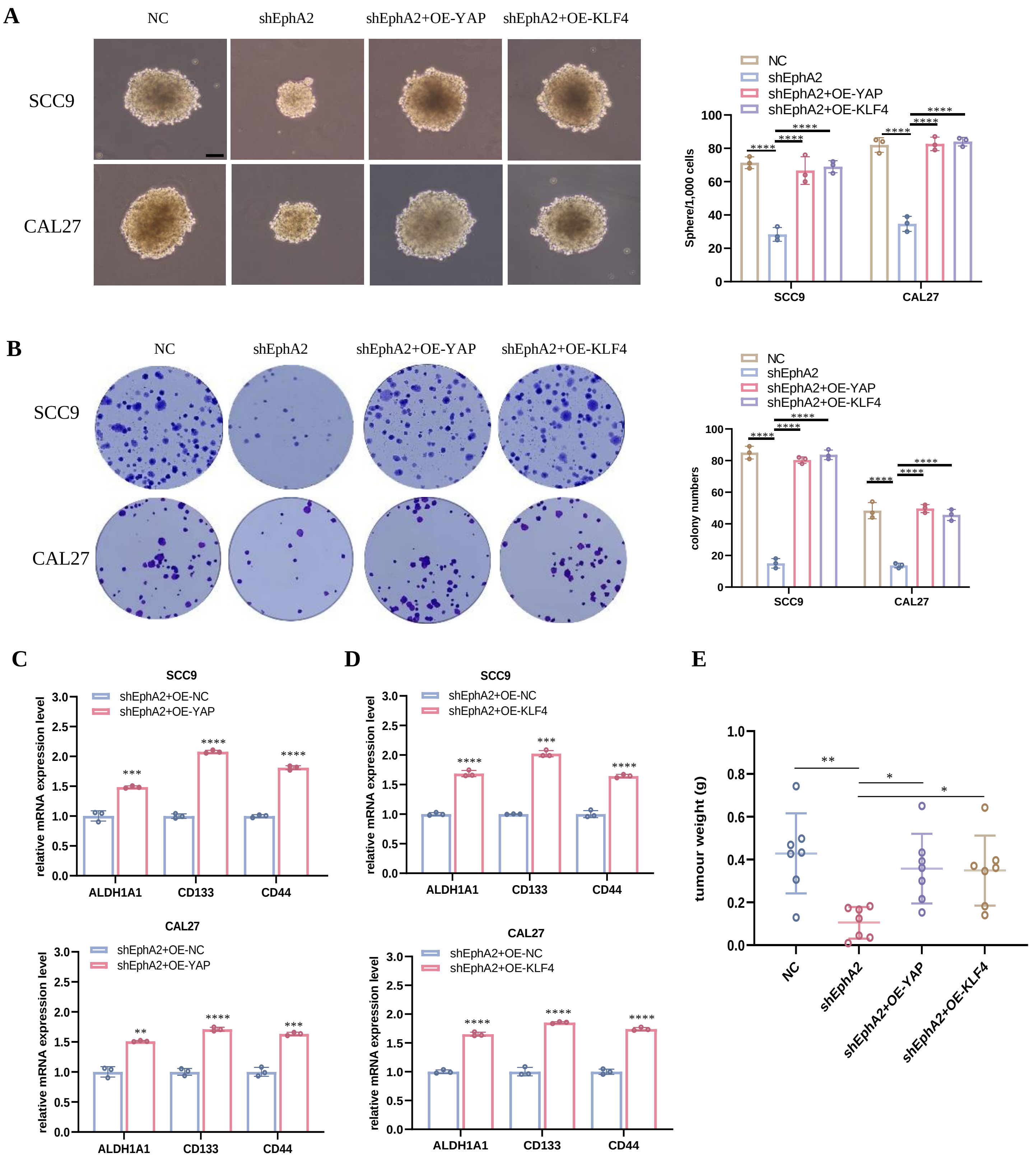

Supplementary Fig. 6 A-D Overexpression of YAP or KLF4 reversed the inhibitory effect of shEphA2 on the sphere-forming (A), and colony-forming abilities (B) and the expression of CSC-related markers (C, D). **E** Weights of tumors excised from the nude mice. n=7.

Supplementary Table 1

Patients	Type	Sex	Age	Location	TNM
1	OSCC	Male	53	Buccal mucosa	T2N0M0
2	OSCC	Male	45	Palate	T2N0M0
3	OSCC	Male	72	Buccal mucosa	T2N0M0
4	OSCC	Female	57	Tongue	T2N0M0
5	OSCC	Male	55	Tongue	T2N1M0
6	OSCC	Male	52	Tongue and Mouth floor	T3N0M0
7	OSCC	Female	66	Tongue	T2N0M0
8	OSCC	Male	66	Tongue and Mouth floor	T4N2bM0
9	OSCC	Male	43	Tongue	T1N0M0
10	OSCC	Female	52	Tongue	T2N0M0
11	OSCC	Male	87	Palate	T1N0M0
12	OSCC	Male	47	Tongue	T2N2M0
13	OSCC	Female	60	Tongue	T1N0M0
14	OSCC	Male	81	Tongue	T2N2bM0
15	OSCC	Male	51	Tongue and Mouth floor	T4N2cM0
16	OSCC	Female	41	Buccal mucosa	T4N0M0
17	OSCC	Male	59	Tongue	T3N1M0
18	OSCC	Male	53	Mouth floor	T2N0M0
19	OSCC	Female	67	Tongue	T2N2M0
20	OSCC	Male	71	Soft Palate	T2N1M0
21	OSCC	Female	68	Buccal mucosa	T2N2M0
22	OSCC	Male	54	Tongue	T2N1M0
23	OSCC	Male	54	Lip	T2N1MO
24	OSCC	Male	56	Gingiva and Mouth floor	T3N0M0
25	OSCC	Male	80	Gingiva	T4N0M0
26	OSCC	Male	61	Gingiva and Mouth floor	T1N0M0
27	OSCC	Male	60	Mouth floor	T4N2bM0
28	OSCC	Male	56	Tongue	T2N0M0
29	OSCC	Female	77	Buccal mucosa and Gingiva	T4N2M0
30	OSCC	Male	50	Tongue	T1N0M0
31	OSCC	Male	60	Buccal mucosa	T1N2M0
32	OSCC	Female	80	Buccal mucosa	T2N0M0
33	OSCC	Male	53	Tongue and Gingiva	T3N1M0
34	OSCC	Female	80	Gingiva	T2N0M0
35	OSCC	Male	68	Tongue	T2N0M0
36	OSCC	Male	71	Soft Palate	T4N0M0
37	OSCC	Female	54	Tongue	T3N1M0
38	OSCC	Male	44	Tongue	T4N2M0
39	OSCC	Female	48	Tongue	T2N1M0
40	OSCC	Male	47	Tongue	T3N0M0
41	OSCC	Female	71	Buccal mucosa	T2N1M0
42	OSCC	Male	45	Palate	T4N0M0
43	OSCC	Male	64	Tongue	T2N0M0
44	OSCC	Female	70	Gingiva	T2N0M0
45	OSCC	Male	60	Buccal mucosa	T3N2bM0
46	OSCC	Male	56	Mouth floor	T4N1M0
47	OSCC	Male	56	Gingiva	T2N0M0
48	OSCC	Female	55	Lip	T2N0M0
49	OSCC	Male	69	Soft Palate	T2N0M0
50	OSCC	Male	62	Gingiva	T4N0M0
51	OSCC	Male	55	Tongue and Mouth floor	T2N1M0
52	OSCC	Male	36	Tongue	T2N0M0
53	OSCC	Female	58	Gingiva	T2N0M0
54	OSCC	Male	63	Tongue	T2N0M0
55	OSCC	Male	76	Tongue	T2N2cM0
56	OSCC	Female	55	Buccal mucosa and Gingiva	T3N0M0
57	OSCC	Male	52	Tongue	T1N0M0
58	OSCC	Female	34	Tongue	T2N0M0
59	Normal	Female	59	Tongue	
60	Normal	Female	43	Tongue	
61	Normal	Male	66	Tongue	
62	Normal	Male	77	Buccal mucosa	
63	Normal	Male	67	Tongue	
64	Normal	Male	62	Buccal mucosa	
65	Normal	Male	49	Palate	
66	Normal	Female	85	Tongue	
67	Normal	Female	59	Tongue	
68	Normal	Male	63	Tongue	
69	Normal	Male	62	Gingiva	
70	Normal	Male	76	Tongue	

Supplementary Table 1. Patient information of tissue microarrays.

Supplementary Table 2

Patients	Type	Sex	Age	Location
1	OSCC	male	73	Tongue
2	OSCC	male	70	Tongue
3	OSCC	male	75	Buccal mucosa
4	OSCC	male	65	Tongue
5	OSCC	male	53	Tongue
6	OSCC	male	69	Tongue and Mouth floor
7	OSCC	male	53	Tongue
8	OSCC	male	50	Mouth floor
9	OSCC	femal	57	Buccal mucosa
10	OSCC	male	48	Buccal mucosa
11	OSCC	male	70	Tongue
12	OSCC	male	39	Tongue
13	OSCC	femal	76	Gingiva
14	OSCC	femal	72	Tongue
15	OSCC	male	55	Tongue and Mouth floor
16	OSCC	male	82	Tongue

Supplementary Table 2. Patient information of Fig.1a (patient 1-8) and Fig.1b (patient 9-16)

Supplementary Table 3

	Forward (5′-3′)	Reverse (5′-3′)
EphA2	TGGCTCACACACCCGTATG	GTCGCCAGACATCACGTTG
YAP	TAGCCCTGCGTAGCCAGTTA	TCATGCTTAGTCCACTGTCTGT
KLF4	ACCTACACAAAGAGTTCCCATC	TGTGTTTACGGTAGTGCCTG
SOX2	GACCAGCTCGCAGACCTACAT	ATGGAGCCAAGAGCCATGC
OCT4	ACATGTGTAAGCTGCGGCC	GTTGTGCATAGTCGCTGCTTG
c-MYC	CCTGGTGCTCCATGAGGAGAC	CAGACTCTGACCTTTTGCCAGG
BMI1	CGTGTATTGTTCGTTACCTGGA	TTCAGTAGTGGTCTGGTCTTGT
NAONG	GAAATACCTCAGCCTCCAGC	GCGTCACACCATTGCTATTC
CD133	AGTCGGAAACTGGCAGATAGC	GGTAGTGTTGTACTGGGCCAAT
ALDH1A1	TGTTAGCTGATGCCGACTTG	TTCTTAGCCCGCTCAACACT
CD44	CTGCCGCTTTGCAGGTGTA	CATTGTGGGCAAGGTGCTATT
TEAD3	ACAGGTGTCCAGCCACATAC	CATGGCCTTGATGCCAACC
GAPDH	GGAGCGAGATCCCTCCAAAAT	GGCTGTTGTCATACTTCTCATGG

Supplementary Table 3. The primer sequence of RT-qPCR

Supplementary Table 4

	Forward (5′-3′)	Reverse (5′-3′)
YAP-siNC	UUCUCCGAACGUGUCACGUTT	ACGUGACACGUUCGGAGAATT
YAP-si1	GCUCAGAUCCUUUCCUUAATT	UUAAGGAAAGGAUCUGAGCTT
YAP-si2	CCUCACAGCAGAACCGUUUTT	AAACGGUUCUGCUGUGAGGTT
YAP-si3	CCAAGUCUGCAGGAAGCUUTT	AAGCUUCCUGCAGACUUGGTT
TEAD3-siNC	UUCUCCGAACGUGUCACGUTT	ACGUGACACGUUCGGAGAATT
TEAD3-si1	GCAAACAGGUGGUAGAGAATT	UUCUCUACCACCUGUUUGCTT
TEAD3-si2	CCAGUGUCCUGCAGAACAATT	UUGUUCUGCAGGACACUGGTT
TEAD3-si3	GGAAGACUCGGACGAGAAATT	UUUCUCGUCCGAGUCUUCCTT

Supplementary Table 4. The sequence of RNA interference assay.

Supplementary Table 5

site	Forward (5′-3′)	Reverse (5′-3′)
270~277	CTAGGTTGACACCAGCCTAAGC	GGTGTTTACTTTGGATTCCGGC
405~412	AAAGTGATACCGAACACCACATTCA	CTCTCCCCTTGGTTTGTGATCAGTA
1049~1056	TGCCACTGCATTCCAGCTTG	TTTCAACCAGCCATCTCGAAGC
1286~1293	ATGCCTCTGAGTAAATCTTGAGCC	GAGAAGGCCAGAGGAGTGTTCC
1981~1988	TTAGCTGCCATAGCAACGATG	CGCGTTCCTTACTTATAACTTCCT

Supplementary Table 5. The primer sequence in ChIP-qPCR
